# Effects of Thermal Treatment on the Physical Properties of Edible Calcium Alginate Gel Beads: Response Surface Methodological Approach

**DOI:** 10.3390/foods8110578

**Published:** 2019-11-15

**Authors:** Seonghui Kim, Chungeun Jeong, Suengmok Cho, Seon-Bong Kim

**Affiliations:** Department of Food Science and Technology, Institute of Seafood Science, Pukyong National University, Busan 48513, Korea; shkim.pknu@gmail.com (S.K.); jeongpknu@gmail.com (C.J.); scho@pknu.ac.kr (S.C.)

**Keywords:** calcium alginate gel, bead, thermal treatment, physical property, response surface methodology

## Abstract

Calcium alginate gel (CAG) has been widely investigated for the development of artificial foods; however, there are few studies on its thermal stability. This study aimed to monitor changes in the physical properties of CAG beads during heat treatment using response surface methodology. Heating temperature (X_1_, 40–100 °C) and heating time (X_2_, 5–60 min) were chosen as independent variables. The dependent variables were rupture strength (Y_1_, kPa), size (Y_2_, μm), and sphericity (Y_3_, %). The heating temperature (X_1_) was the independent variable that had a significant effect on the rupture strength (Y_1_) and size (Y_2_). Rupture strength (Y_1_) increased as the heating temperature (X_1_) increased; at the same time, the CAG beads size (Y_2_) decreased. With all conditions, the values of sphericity (Y_3_) were over 94%. SEM images revealed that increase in the rupture strength of the CAG beads by heat treatment resulted from their porous structures. Loss of moisture by syneresis, occurring with heat treatment, was judged to create a dense porous structure of CAG beads. Our findings offer useful information for cooking or sterilizing food products utilizing CAG beads. In addition, thermal treatment could be applied to produce hard CAG beads with a high rupture strength.

## 1. Introduction

Alginate is a linear anionic polysaccharide derived from brown seaweeds and is composed of (1-4)-linked β-D-mannuronic (M) and α-L-guluronic acid (G) residues [1,2]. It can form gel through cross linking with divalent cations, forming an egg-box structure [1,3]. Among divalent cations, calcium is the most commonly used cation for ionotropic gelation of alginate [1]. Calcium alginate gel (CAG) is easily produced by extrusion methods, by dripping the alginate solution into a calcium ion solution [4]. CAG has been investigated as a thickener, stabilizer, and restructuring agent in food processing because of its unique gelling abilities [5]. Furthermore, CAG has been applied in cell encapsulation, drug delivery and tissue engineering [6].

In recent years, there has been an increasing interest in CAG as a biomaterial for making artificial or imitative foods. Some researchers have studied the optimization of the processing of fish roe [7,8] and effects of the physicochemical parameters of cooked rice [9] analogs using CAG. Many studies have focused on optimizing the processing conditions, such as concentration of the alginate or calcium and gelation time. To date, little attention has been paid to the thermal stability of CAG. In the food industry, foods prepared with CAG are subjected to various forms of heat treatment. For example, food safety issues require thermal processes for sterilization of the CAG; moreover, CAG foods may be heated for cooking or manufacturing. During thermal treatments, CAG’s physical properties undergo changes; however, no information can be found. The physical properties of alginate gels are used to enhance food product quality and stability during storage [10,11]. Therefore, it is important to investigate the effects of thermal treatment on physical property changes in CAG, for application and processing of CAG-based foods.

This study aimed to understand the effects of heat treatment on the physical properties of CAG beads for its application to fish roe analogs. The central composite design (CCD) of response surface methodology (RSM) was adopted to monitor the effects of heat treatment. RSM is a statistical procedure frequently used for optimization and monitoring of food processes. The basic principle of RSM is to describe model equations defining the effect of test variables on responses and determine interrelationships among test variables in any response [12]. The CCD was reported for designing the experiment, to create a model, and to optimize the process variables with sensory and hedonic properties of food products [13]. Heating temperature and time were chosen as the independent variables. Rupture strength, size, and sphericity were measured to explore the physical property changes of the CAG beads after heat treatment. Furthermore, microstructures were analyzed using scanning electron microscopy.

## 2. Materials and Methods

### 2.1. Materials

Sodium alginate (Junsei Chemical Co., Ltd., Tokyo, Japan) and calcium lactate (Daejung chemical & metal Co., Ltd., Siheung, Korea) were used as the functional materials for CAG beads. All chemicals and reagents used in this study were of an analytical grade.

### 2.2. Preparation of CAG Beads

**Figure 1:**
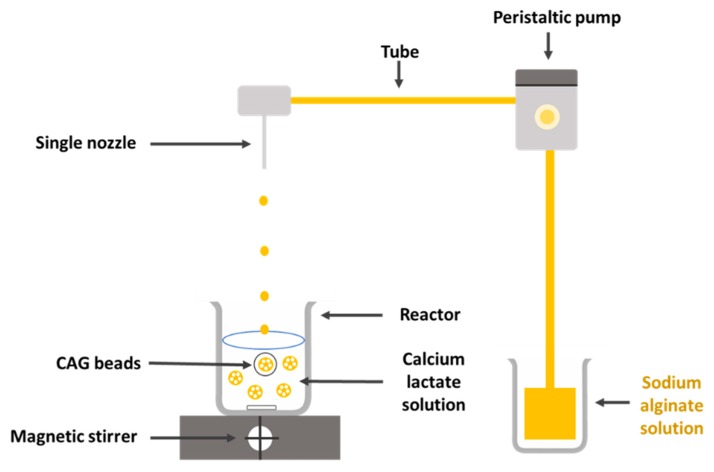
Simple schematic diagram for the preparation of calcium alginate gel (CAG) beads using a single nozzle.

A sodium alginate solution (2.57%, *w*/*v*) was dropped through a single nozzle 20G (inner diameter: 0.60 mm, outer diameter: 0.90 mm) using a peristaltic pump (Cassette tube pump SMP-23, Eyela, Tokyo, Japan) into a calcium lactate solution (1.52%, *w*/*v*). The stirring speed of calcium lactate solution (250 mL) in the reactor (500 mL) was 300 rpm. The distance between the nozzle tip and the surface of the calcium lactate solution was 8 cm. The allowed gelation time was 20 min. The CAG beads were rinsed with distilled water to remove any remaining calcium lactate. A schematic diagram for preparation of CAG beads is shown in Figure 1.

### 2.3. Measurement of Size and Sphericity of CAG

CAG beads sizes were measured by a stereomicroscope (Olympus SZX16; Tokyo, Japan) and were represented as diameter (mm). Sphericity (%) was determined by the percent ratio of the minor diameter to major diameter, obtained from the size measurements; five beads were randomly selected from each experimental condition for measurement.

### 2.4. Measurement of Rupture Strength

The rupture strength was measured using a rheometer (Model CR-100D, Sun Scientific Co., Ltd., Tokyo, Japan) with the following conditions: plunger diameter, 25 mm; penetration speed, 80 mm/min; adaptor area, 4.91 cm^2^; and load cell force, 0.1 kN. Five samples were measured for each experiment.

### 2.5. Experimental Design

To monitor the effects of heat treatment on the physical properties of the CAG beads, a central composite design (CCD) was adopted in the optimization of the CAG beads. CCD in this design comprises 2^2^ factorial points, 4 axial points (α = 1.414), with 3 replicates of the central points. Heating temperature (X_1_, °C) and time (X_2_, min) were chosen as independent variables. The range and center point values of the two independent variables were based on the results of preliminary experiments (Table 1). The dependent variables were rupture strength (Y_1_, kPa), size (Y_2_, μm), and sphericity (Y_3_, %), indicating the physical characteristics of the CAG beads. The experiments were randomized to minimize the effects of unexpected variability in the observed responses.

### 2.6. Data Analysis and Optimization

Using the response surface methodology of MINTAB statistical software (Version 16, Minitab Inc., Harrisburg, PA, USA), Equation (1) was used to fit results [14].

(1) $$Y=\beta_{0}+\overset{4}{\underset{i=1}{\sum }}\beta_{i}X_{i}+\overset{4}{\underset{i=1}{\sum }}\beta_{ii}X_{i}^{2}+\overset{3}{\underset{i=1}{\sum }}\overset{4}{\underset{j=i+1}{\sum }}\beta_{ij}X_{i}X_{j}$$

Here, *Y* is a dependent variable (rupture strength, size, or sphericity), *β_0_* is a constant, *β_i_*, *β_ii_, β_ij_* are regression coefficients, *X_i_* and *X_j_* are levels of the independent variables. The target value of Y_1_ maximum and response optimization were calculated by the response optimizer in the MINITAB software. Three-dimensional response surface plots were developed using Maple software (Version 7, Waterloo Maple Inc., Waterloo, Ontario, Canada) and represented a function of two independent variables.

### 2.7. Scanning Electron Microscopy (SEM)

To investigate the influence of heat treatment on CAG bead microstructure, the CAG beads were immersed in liquid nitrogen and cut with a knife to obtain the cross section. The CAG beads then underwent lyophilization in a freeze-dryer (CoolSafe, Lynge, Denmark) for 24 h. Moreover, the CAG beads were fixed to a sample with a gold layer using an ion sputter device (Hitachi, E-1010, Tokyo, Japan), and viewed by SEM (JSM-6490LV, JEOL Ltd., Tokyo, Japan) at an accelerating voltage of 15 kV.

### 2.8. Weight and Water Contents of the CAG Beads

Water content (%) was measured with a moisture analyzer (MX-50, A&D, Tokyo, Japan). The CAG beads were weighed and placed on the analyzer and heated at 100 °C until no weight change was observed. The difference between the original and final weight was considered as the water content. Weighing was performed on a digital balance (Model Radwag, AS 220-R1, Radom, Poland). Water content and weight are expressed as the mean of three replications.

### 2.9. Measurement of Density

The mean weights and diameters of the beads were measured and used to calculate densities of beads using the following Equation (2):

(2) $$D=\frac{M}{V},and\;V=\frac{4}{3}\;\pi r^{3}$$

where *D* is the density of the beads; *M* is the weight of the beads; *V* is the volume of the beads; *r* is the radius of the beads.

## 3. Results and Discussion

### 3.1. Diagnostic Checking of the Fitted Models

Table 2 presents the experimental design and values of the dependent variables considering different heat treatment conditions. It is necessary to fit the quadratic polynomial equation to describe the behavior of the dependent variables on independent variables [15]. Response surface model equations are estimated by a statistical approach called the least-squares technique [16]. The fitted response surface model equations are shown in Table 3. The determination coefficient (*R*^2^) value indicates that the model equations described the experimental designs adequately [17]. In general, the more suitable the consideration of the lowest standard deviation, the highest *R*-squared values (*R*^2^, adjusted *R*^2^), the better the fit [18]. A *p*-value smaller than 0.05 implies that the corresponding model term is significant [19]. The *R*^2^ values of Y_1_ (rupture strength), Y_2_ (size), and Y_3_ (sphericity) were 0.904, 0.888 (*p* < 0.05), and 0.935 (*p* < 0.01), respectively. The statistical significance of the quadratic polynomial model equation was evaluated by an analysis of variance (ANOVA), shown in Table 4; the results for the models show the response of the three dependent variables. The results of the lack of fit test, which indicates the fitness of the model [20], show that the *F*-values of Y_1_, Y_2_, and Y_3_ were 9.57, 8.63, and 1.57, respectively; the related p-values were not significant (0.096, 0.106, and 0.412, respectively) (*p* > 0.05). These results indicate that the models were suitable for accurately predicting variation [21].

### 3.2. Response Surface Plots and the Effect of Factors

**Figure 2:**
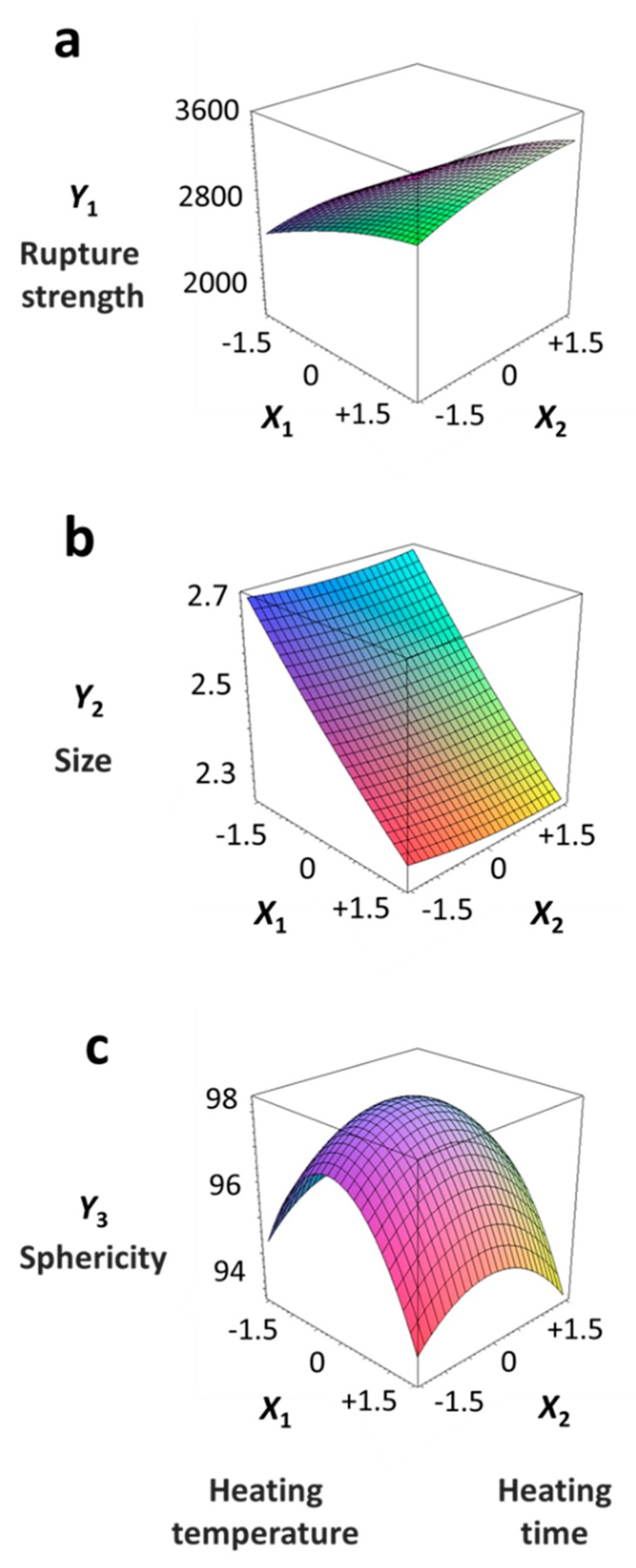
Three-dimensional response surface plots for rupture strength (**a**), size (**b**), and sphericity (**c**). X_1_; Heating temperature (°C), X_2_; Heating time (min).

Table 5 provides the calculated data for significance with t-statistic and the estimated coefficients of the linear (X_1_, X_2_), quadratic (X_1_X_1_, X_2_X_2_), and interaction (X_1_X_2_) terms for the three dependent variables (Y_1_, Y_2_, and Y_3_). The effects of heating temperature (X_1_) and heating time (X_2_) on rupture strength (Y_1_), size (Y_2_), and sphericity (Y_3_) are expressed as a three-dimensional plot (Figure 2). The larger t-value and smaller p-value indicate the significance of parameters [22].

The rupture strength (Y_1_) increased when the heating temperature (X_1_) increased from 40 °C (−1.414) to 100 °C (+1.414); Y_1_ decreased when heating time (X_2_) increased from 5 min (−1.414) to 60 min (+1.414) (Figure 2a). The linear term for Y_1_ was significant (*p* < 0.01), while their square terms and interaction terms were not significant (*p* > 0.05) (Table 4). The X_1_ term for Y_1_ was significant (*p* < 0.01), while the X_2_ term for Y_1_ was not significant (*p* > 0.05) (Table 5). We found that the heating temperature (X_1_) is a significant factor affecting the rupture strength (Y_1_); however, there was no statistically significance between rupture strength (Y_1_) and heating time (X_2_). This result supports previous study [23], which studied the effect of temperature on the structure of calcium alginate beads. This study shows that the comparison of the mechanical resistance at different temperatures (80, 110, and 130 °C), for the same period (0, 5, 10, 15, and 20 min) of incubation, shows that a higher resistance is always obtained at the higher temperature. A previous study also reported that the hardness of the alginate-guar gels mixed with pimiento pulp significantly increased when applying thermal treatment (80 °C for 15 min) [24]. The authors reported that the increase in hardness of the alginate-guar gels originated from the occurrence of gel shrinkage, making them more compact during heat treatment [24].

The size (Y_2_) sharply decreased with an increase in the heating temperature (X_1_) from 40 °C (−1.414) to 100 °C (+1.414); however, there was no significant relationship between size (Y_2_) and heating time (X_2_) from 5 min (−1.414) to 60 min (+1.414) (*p* > 0.05) (Figure 2b). The linear terms for Y_2_ were significant (*p* < 0.01), while their square terms and interaction terms were not significant (*p* > 0.05) (Table 4). The X_1_ term for Y_2_ was significant (*p* < 0.01), while X_2_ term for Y_2_ was not significant (*p* > 0.05) (Table 5). Here, it is apparent that heating temperature (X_1_) is a significant factor affecting the size (Y_2_); however, there was no significant relationship between the size (Y_2_) and heating time (X_2_). This finding is consistent with previous study results [25], where the average bead size, after 30 min of heat treatment (90 °C), was showing 0.3 mm (3.62 mm to 3.32 mm) of shrinkage. Likewise, other authors [23] found that the reduction in bead size clearly depends on both time (0, 5, 10, 15, and 20 min) and temperature (80, 110, and 130 °C). The size reduction may be related to water loss [26,27,28]. As the CAG beads were heated, there was a greater amount of water exuded and therefore, greater the size reduction.

The sphericity (Y_3_) increased as the coded values of independent variables approached zero (Figure 2c). The linear and interaction terms for Y_3_ were not significant (*p* > 0.05), while their square terms were significant (*p* < 0.01) (Table 4). The linear and interaction terms for Y_3_ were not significant a (*p* > 0.05), while their square terms were significant (*p* < 0.01) (Table 5). Previous authors [7] found that caviar analogs using CAG had sphericities ranging from 90% to 100%, measured with a digital microscope, and could not be differentiated with the naked eye. In Figure 2, the CAG beads after heat treatment from 40 °C (−1.414) to 100 °C (+1.414) showed sphericities ranging from 94% to 97%. We believe, therefore, that the change of heating temperature and heating time did not significantly affect the sphericity of the CAG beads, as they retained their spheroid shape.

In conclusion, the rupture strength (Y_1_) and size (Y_2_) affected dependent variables, with heating temperature (X_1_) being the most important factor. In addition, as the CAG beads’ heating temperature (X_1_) increased, the rupture strength (Y_1_) increased and the size (Y_2_) decreased.

### 3.3. Microstructure

**Figure 3:**
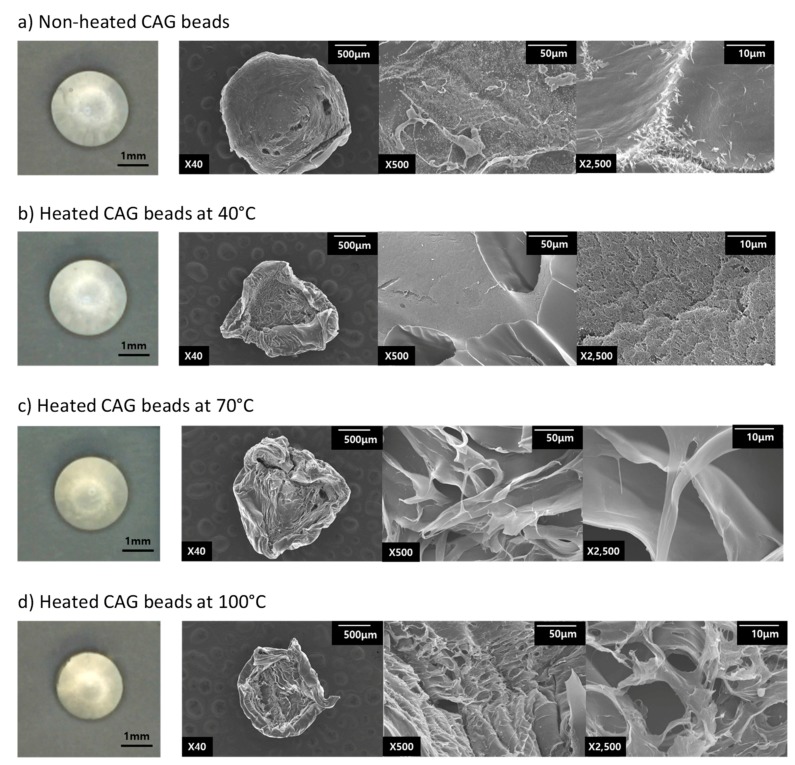
Digital microscope (left), and SEM (right) photographs of CAG beads frozen in liquid nitrogen and freeze-dried: (**a**) before heat treatment CAG bead; (**b**) CAG beads were heated at 40 °C; (**c**) CAG beads were heated at 70 °C; (**d**) CAG beads were heated at 100 °C. Magnification of the images are 40×, 500×, and 2500×.

To better understand the effects of heat treatment on physical properties of CAG beads, we analyzed the microstructure of the CAG beads through SEM image. For this, freeze-dried CAG beads were used and were compared with CAG beads heated at 40, 70, and 100 °C (Figure 3). Digital microscope observations showed that in both the unheated and heated tests, the CAG beads retained their spheroid shape. However, the freeze-dried CAG beads did not retain their spheroid shape. We believe that the water loss during freeze-drying involves shrinkage and deformation of CAG beads dimension, which increases the particle density [29]. The CAG beads after heat treatment presented an altered structure when compared to unheated CAG beads’ structure. Unheated CAG beads are homogeneous and smooth (Figure 3a) because they did not lose water. Conversely, after heat treatment, the CAG beads have a greater porous structure (Figure 3b–d) because they lost water. When the heating temperature (*X*_1_) increased from 70 °C to 100 °C, the CAG beads gradually formed a more compact gel network with a homogeneous distribution of small pores, while the CAG beads, after heat treatment at 40 °C exhibited void pores. The change of microstructure in the CAG beads could explain the change in physical properties. Our findings reveal that at higher temperatures, the rupture strength increased (Table 2). This indicates that water loss leads to a more dense porous structure, thus increasing rupture strength. In Table 6, the density of unheated CAG beads and that of heated CAG beads at 40, 70, and 100 °C was calculated. These findings provide evidence that the increase in rupture strength was due to an increase in the density [30]. A similar microstructure was observed previously [31] and a higher strength of gel microstructure was more uniform and continuous with smaller voids.

**Figure 4:**
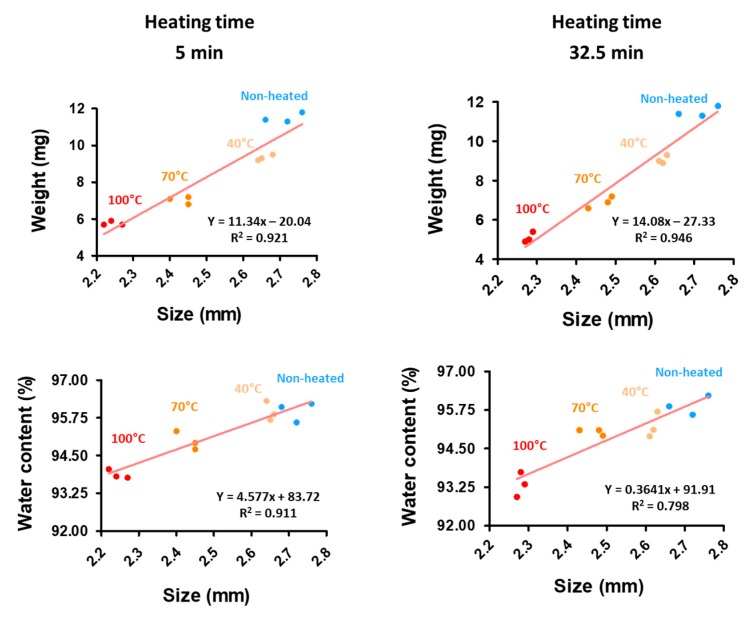
Correlation between weight (mg) and size (mm), water content (%), and size (mm) at 5 min; and weight (mg) and size (mm), water content (%), and size (mm) at 32.5 min.

Heating at 70 and 100 °C significantly reduced the CAG bead size believed to result from water loss [26,27,28]. Thus, we believe that syneresis, a phenomenon whereby water molecules are exuded out of a gel matrix because of an external force that contracts the gel, occurs when treated with heat [11,32]. We investigated whether this size reduction was correlated to water leakage, water content, size, and weight of the CAG beads after heat treatment at 40, 70, and 100 °C for 5 min to 32.5 min (Figure 4). The size (Y_2_) and weight were correlated (*R*^2^ = 0.921, 0.946). Here, data show that the higher the heated temperature of the CAG beads, the more significant the size (Y_2_) and weight reduction. Moreover, size (Y_2_) and water content were correlated (*R*^2^ = 0.911, 0.798). However, the water content of the CAG beads decreased as the heat treatment increased. The weight of the non-heated CAG beads was 11.5 mg and the weight of the heated CAG beads at 100 °C was 5.8 mg. The water content of the non-heated CAG beads was 95.9%, while that of the CAG beads heated at 100 °C was 93.3%. Our findings reveal that the absolute water content 11.03 mg was reduced to 5.39 mg and there was a 51.2% water loss.

In conclusion, the SEM images, with the water content, weight, and size correlations show that the denser the structure of the CAG beads after exposure to higher heating temperatures, the less available space there is for water, resulting in an increased rupture strength and reduced size.

### 3.4. Optimal Conditions and Verification

In the food industry, it is recommended that minimum changes occur in foods prepared with CAG during thermal processes such as sterilization, cooking, and manufacturing. Therefore, in this study, three optimized heat-treated CAG beads were prepared to give similar results as non-heated CAG beads considering the rupture strength (Y_1_), size (Y_2_), and sphericity (Y_3_) and were verified (Table 7). The optimal conditions, including the coded and uncoded values of the independent variables, are shown in Table 8. The optimal conditions indicated that the rupture strength (Y_1_), size (Y_2_), and sphericity (Y_3_) of the unheated CAG beads were 3450 ± 112.47 kPa, 2.60 ± 0.05 mm, and 96.5 ± 2.14%, respectively. According to the results of CCD, the optimal conditions of X_1_ (heating temperature) and X_2_ (heating time) were −0.6649 (56.0371 °C) and −1.414 (5 min), respectively. To facilitate the operation, the optimal process conditions of heating time and temperature were 56 °C and 5 min, respectively. The predicted values of Y_1_, Y_2_, and Y_3_ for each optimal condition were 2993 kPa, 2.60 mm, and 96.8%, respectively. To verify the accuracy of the predicted Y_1_, Y_2_, and Y_3_ values, the CAG beads were prepared under each of the optimal conditions and tested. Percentage error evaluation is helpful in establishing the validity of generated model equations and also to describe the domain of applicability of the optimization model [33]. The experimental values of Y_1_, Y_2_, and Y_3_ were 2844 ± 66.64 kPa, 2.55 ± 0.02 mm, and 96.0 ± 2.25%, respectively, similarly to the predicated values, with error values of 4.98, 1.92, and 0.83, respectively (Table 9).

## 4. Conclusions

Calcium alginate gel (CAG) is used to make artificial or imitative foods because of its unique gelling abilities and is a promising biomaterial in the food industry. In the food industry, it is important to verify stability and process suitability for artificial or imitative foods using CAG; the effects of heat treatment on the physical properties of CAG beads can be utilized in several ways. Our findings reveal that the heating temperature (X_1_) was the factor that had the greatest effect on the rupture strength (Y_1_) and size (Y_2_). The CAG beads increased in rupture strength and decreased in size as the heating temperature increased because of water loss. RSM was successfully employed to optimize the non-heated CAG beads. Under each optimal condition, Y_1_, Y_2_, and Y_3_ were 2844 ± 66.64 kPa, 2.55 ± 0.02 mm, and 96.0 ± 2.25%, respectively. Our results indicate that the heat treatment of CAG can be used not only for sterilization and cooking but also as a processing technique by controlling the physical properties.

## Figures and Tables

**Table 1:** Experimental range and values of independent variables in the central composite design for monitoring the effects of thermal treatment on the physical properties of CAG beads.

Independent Variables	Symbol	Range and Levels				
		–1.414	–1	0	+1	+1.414
Heating temperature (°C)	X_1_	40	49	70	91	100
Heating time (min)	X_2_	5	13	33	52	60

**Table 2:** Central composite design matrix and values of dependent variables for monitoring the effects of thermal treatment on the physical properties of CAG beads.

Run No.		Independent Variables				Dependent Variables *		
		Coded Values		Uncoded Values				
		X_1_	X_2_	X_1_	X_2_	Y_1_	Y_2_	Y_3_
Factorial portions	1	–1	–1	49	13.1	2658	2.73	96.6
	2	1	–1	91	13.1	3692	2.31	95.6
	3	–1	1	49	52	2243	2.73	96.6
	4	1	1	91	52	3516	2.28	95.5
Axial portions	5	–1.414	0	40	32.5	2597	2.62	96.0
	6	1.414	0	100	32.5	3408	2.28	95.4
	7	0	–1.414	70	5	3244	2.46	97.6
	8	0	1.414	70	60	2773	2.44	96.7
Center points	9	0	0	70	32.5	3060	2.48	98.0
	10	0	0	70	32.5	3177	2.43	98.2
	11	0	0	70	32.5	3032	2.49	98.7

X_1_: Heating temperature (°C), X_2_: Heating time (min). Y_1_: Rupture strength (kPa), Y_2_: size (mm), Y_3_: sphericity (%) * Each experiment was performed five times and the average value is used here.

**Table 3:** Response surface model equations for monitoring the effects of thermal treatment on the physical properties of CAG beads.

Quadratic Polynomial Model Equations	*R* ^2^	Adj *R*^2^	S	*p*-Value
Y_1_ = 3090 + 431.7 X_1_ – 157.1 X_2_ – 38.1 X_1_^2^ – 35.1 X_2_^2^ + 59.8 X_1_X_2_	0.904	0.808	190.633	0.014
Y_2_ = 2.34667 – 0.1689 X_1_ – 0.0073 X_2_ – 0.0073 X_1_^2^ – 0.0073 X_2_^2^ – 0.0075 X_1_X_2_	0.888	0.777	0.0759091	0.020
Y_3_ = 98.300 – 0.369 X_1_ – 0.172 X_2_ – 1.388 X_1_^2^ – 0.663 X_2_^2^ – 0.025 X_1_X_2_	0.935	0.870	0.417781	0.005

X_1_: Heating temperature (°C), X_2_: Heating time (min). Y_1_: Rupture strength (kPa), Y_2_: size (mm), Y_3_: sphericity (%).

**Table 4:** Analysis of variance for dependent variables.

Dependent Variables	Sources	DF	SS	MS	*F*-Value	*p*-Value
Y_1_ Rupture strength (kPa)	Regression					
	Linear	2	1688737	84436	23.23	0.003 *
	Square	2	11756	5878	0.16	0.855
	Interaction	1	14280	14280	0.39	0.558
	Residual					
	Lack of fit	3	169872	56624	9.57	0.096
	Pure error	2	11833	5916		
	Total	10	1896479			
Y_2_ Size (mm)	Regression					
	Linear	2	0.228518	0.114259	19.83	0.004 *
	Square	2	0.000464	0.000232	0.04	0.961
	Interaction	1	0.000225	0.000225	0.04	0.851
	Residual					
	Lack of fit	3	0.026744	0.008915	8.63	0.106
	Pure error	2	0.002067	0.001033		
	Total	10	0.258018			
Y_3_ Sphericity (%)	Regression					
	Linear	2	1.3223	0.6611	3.79	0.100
	Square	2	11.2716	5.6358	32.29	0.001 *
	Interaction	1	0.0025	0.0025	0.01	0.909
	Residual					
	Lack of fit	3	0.6127	0.2042	1.57	0.412
	Pure error	2	0.2600	0.1300		
	Total	10	13.4691			

DF: Degrees of freedom, SS: Sum of square, MS: Mean square, * Significant at *p* < 0.05.

**Table 5:** Estimated coefficients of the fitted quadratic polynomial equations for dependent variables based on the *t*-statistic.

**Parameters**	**Y_1_** **Rupture Strength (kPa)**			
	**Coefficient**	**Square Error**	***t*-Value**	***p*-Value**
Constant	3090	110	28.07	0.001
X_1_	431.7	67.4	6.41	0.001 *
X_2_	–157.1	67.4	–2.33	0.067
X_1_X_1_	–38.1	80.2	-0.48	0.654
X_2_X_2_	–35.1	80.2	–0.44	0.680
X_1_X_2_	59.8	95.3	0.63	0.558
**Parameters**	**Y_2_** **Size (mm)**			
	**Coefficient**	**Square Error**	***t*** **-Value**	***p*-Value**
Constant	2.4667	0.0438	56.28	0.001
X_1_	–0.1689	0.0268	–6.29	0.001 *
X_2_	–0.0073	0.0268	–0.27	0.797
X_1_X_1_	0.0073	0.0319	0.23	0.828
X_2_X_2_	0.0073	0.0319	0.23	0.828
X_1_X_2_	–0.0075	0.0380	–0.20	0.851
**Parameters**	**Y_3_** **Sphericity (%)**			
	**Coefficient**	**Square Error**	***t*-Value**	***p*-Value**
Constant	98.300	0.241	407.54	0.001
X_1_	–0.369	0.148	–2.50	0.055
X_2_	–0.172	0.148	–1.16	0.298
X_1_X_1_	–1.388	0.176	–7.89	0.001 *
X_2_X_2_	–0.663	0.176	–3.77	0.013 *
X_1_X_2_	–0.025	0.209	–0.12	0.909

X_1_: Heating temperature (°C), X_2_: Heating time (min), * Significant at *p* < 0.05.

**Table 6:** Density (g/cm^3^) of CAG beads.

Heating Temperature (°C)	Before Heat Treatment	40 °C	70 °C	100 °C
Density (g/cm^3^)	1.17 ± 0.07	1.02 ± 0.03 *	1.04 ± 0.04 *	1.26 ± 0.05

* *p* < 0.05 compared to the before heat treatment (Dunnett’s test).

**Table 7:** The rupture strength, size, and sphericity of before heat treatment CAG beads.

	Y_1_ Rupture Strength (kPa)	Y_2_ Size (mm)	Y_3_ Sphericity (%)
Before heat treatment	3450 ± 112.50	2.60 ± 0.05	96.5 ± 2.15

**Table 8:** Response optimization for processing a heated CAG beads similar result to non-heated CAG beads conditions.

Optimal Conditions		X_1_ Heating Temperature (°C)		X_2_ Heating Time (min)	
		Coded Value	Actual Value	Coded Value	Actual Value
		–0.665	56.0	–1.414	5
Y_1_ Rupture strength (kPa)	Target value	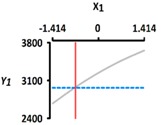		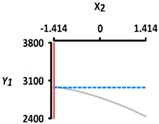	
	3450				
Y_2_ Size (mm)	Target value	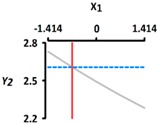		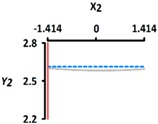	
	2.60				
Y_3_ Sphericity (%)	Target value	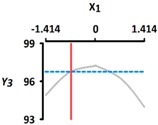		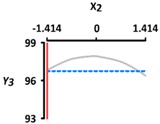	
	96.5				

**Table 9:** Experimental and predicted results of verification under optimized conditions.

	Y_1_ Rupture Strength (kPa)	Y_2_ Size (mm)	Y_3_ Sphericity (%)
Predicted values	2993	2.60	96.8
Experimental values	2844 ± 66.64	2.55 ± 0.02	96.0 ± 2.25
Error (%)	4.98	1.92	0.83

Optimized conditions: heating temperature = 100 °C; heating time = 31 min. Error (%) = [Difference between predicted value and actual value/Predicted value] × 100.
